# Potato Elicitor Peptide StPep1 Enhances Resistance to *Phytophthora infestans* in *Solanum tuberosum*

**DOI:** 10.3390/jof11120893

**Published:** 2025-12-18

**Authors:** Alexander Skripnikov, Tatiana Suprunova, Natalia O. Kalinina, Michael Taliansky

**Affiliations:** 1 Shemyakin–Ovchinnikov Institute of Bioorganic Chemistry, Russian Academy of Sciences, Miklukho-Maklaya Street 16/10, Moscow 119997, Russia; kalinina@belozersky.msu.ru (N.O.K.);; 2 Department of Biology, Lomonosov Moscow State University, Leninskiye Gory 1, Building 12, Moscow 119234, Russia; 3 Doka-Gene Technologies Ltd., Moscow Region, Rogachevo 141880, Russia; 4 A.N. Belozersky Institute of Physico-Chemical Biology, Lomonosov Moscow State University, Leninskiye Gory 1, Building 40, Moscow 119234, Russia

**Keywords:** plant peptides, elicitor peptides, potato, *Phytophthora infestans*

## Abstract

Plant peptides represent a novel molecular tool in crop science due to their essential regulatory roles in plant growth, development, and responses to biotic and abiotic stresses. Although numerous bioactive plant peptides have been identified, a major gap remains in translating these discoveries into practical strategies for crop protection. Synthetic peptides are increasingly recognized as promising biological agents for enhancing crop productivity and protection in an environmentally sustainable manner. In this study, we demonstrate that the potato elicitor peptide StPep1, applied as a foliar spray at nanomolar concentrations (10–100 nM), strongly enhances resistance to the oomycete pathogen *Phytophthora infestans* in *Solanum tuberosum* cv. Gala under controlled climate chamber conditions. Preventive treatment 24 h prior to inoculation markedly reduced disease symptoms, with treated plants exhibiting a phenotype comparable to uninoculated controls. These findings highlight the potential of low-dose StPep1 as an environmentally friendly and cost-effective bioprotective agent, providing a foundation for future translational research and small-scale agricultural applications.

## 1. Introduction

Plant peptides constitute an emerging area in plant biology, owing to their pivotal roles in the regulation of growth, development, and responses to environmental stress [1,2,3]. Synthetic peptides are regarded as promising biological agents with the potential to enhance crop growth and protection in an environmentally sustainable manner, helping to reduce the reliance on chemical pesticides [4,5]. Elicitor peptides differ fundamentally from conventional pesticides, acting as signaling molecules effective at low concentrations, and are generally non-toxic chemical agents by nature. Elicitor peptides such as systemins and Peps, unlike antimicrobial peptides, are not aimed at toxic pathogen management but rather at triggering systemic defense reactions in plants in response to pathogen or pest attack. Pioneers of the discovery of the first plant peptide, systemin [6], subsequently identified the 23-amino acid elicitor peptide AtPep1 from Arabidopsis leaf extracts through bioassay-guided purification, using medium alkalinization in tobacco suspension cultures as a functional assay [7]. Plant elicitor peptides (Peps) represent a widely conserved peptide family found across numerous families of higher plants. Although Pep peptides have not yet been identified as distinct molecules by mass spectrometry, studies in crops such as maize and potato have explored putative Pep peptides using synthetic 23-amino acid peptides based on the AtPep1 sequence originally identified in Arabidopsis [8,9].

The protective activity of synthetic elicitor peptides has been demonstrated in several plant models. For instance, treatment of *Arabidopsis thaliana* with AtPep1 enhanced resistance to the oomycete *Pythium irregulare* and suppressed the growth of the bacterial pathogen *Pseudomonas syringae* pv. *tomato* DC3000 [10].

In maize, application of the synthetic peptide ZmPep1 triggered immune responses that conferred resistance to southern leaf blight and anthracnose stalk rot, caused by *Cochliobolus heterostrophus* and *Colletotrichum graminicola*, respectively [8]. In rice, OsPep3 was shown to activate broad-spectrum defenses against multiple threats, including the pathogenic rice insect brown planthopper (*Nilaparvata lugens*), the fungal pathogen *Magnaporthe oryzae*, and the bacterial pathogen *Xanthomonas oryzae* pv. *oryzae* [11].

Evidence from bananas further underscores the potential of Pep-type peptides in crop protection. Foliar application of putative 23-amino acid peptide MaPep1 and MbPep1 to young potted plants significantly improved resistance to the bacterial pathogen *Ralstonia syzygii* [12]. While the authors highlighted the practical relevance of these peptides, they also noted barriers to immediate agricultural implementation—primarily the high cost of synthetic peptide production. This concern may stem from the relatively elevated concentrations required in their experiments (200 μM), which exceed those typically effective for signaling peptides in other plant systems [13,14]. Cost-related limitations of elicitor peptide applications may be overcome by reducing the effective concentrations to extremely low levels, at which signaling peptides naturally exert their biological activity [6,7,8,9,13,14].

Potato peptide elicitor StPep1 is of significant applied interest as a signal inducer of potato defense reactions. Model experiments have shown that treatment of potato plants with the elicitor peptide StPep1 at low concentrations (100 nM–1 μM) enhances resistance to nematodes *in vitro*, as demonstrated by increased tolerance of Russet Burbank potato plants artificially infected with the phytopathogenic nematode *Meloidogyne chitwoodi* [9].

In our experiments, we demonstrated that foliar application of the StPep1 peptide at nanomolar concentrations to young potato plants under controlled climate chamber conditions enhances resistance to *Phytophthora infestans* (Mont.) de Bary following artificial pathogen inoculation. The oomycete *P. infestans*, is one of the most devastating plant pathogens that causes the severe disease of potato plants referred to as late blight, and accounts for annual substantial yield losses worldwide [15]. Current control measures in the potato industry rely heavily on chemical fungicides, which pose increasing risks to human health and the environment [16]. Therefore, alternative more eco-friendly biotechnological approaches are highly required to reduce toxicity, increase target specificity and provide environmental sustainability. One such promising approach may be based on the application of antimicrobial and immune-inducing peptides. This highlights the urgent need for safer, more sustainable alternatives. Immune-inducing peptides such as StPep1 offer a promising biotechnology-based solution. Importantly, our findings show that StPep1 is effective at extremely low (nanomolar) concentrations, making it not only biologically active but also potentially cost-effective for agricultural use. This study represents a first step toward the development of peptide-based protective treatments for crops and lays the groundwork for subsequent small-scale field trials evaluating StPep1 as a novel, eco-friendly agent for potato disease management.

## 2. Materials and Methods

### 2.1. Plant Material

*In vitro*-propagated *Solanum tuberosum* cv. Gala plants (breeding of Norika GmbH, Groß Lüsewitz, Germany) were used to evaluate the effect of the peptide on potato resistance to *P. infestans*. This cultivar is known for its high susceptibility to late blight, making it a suitable model for assessing disease resistance. We used healthy *in vitro* material free from viral, fungal, and bacterial pathogens. Mini-tubers free from viral, fungal and bacterial infections were planted in a peat-based substrate and grown under controlled conditions. The plants were acclimated before experimental treatments. Experiments were independently repeated three times using plants grown for 3 weeks after transplantation. Each treatment group included five biological replicates (*n* = 5).

### 2.2. Pathogen Inoculation

The *P. infestans* strain VZR ViR21a (obtained from the FSBSI VIZR collection, St. Petersburg, Pushkin, Russia), originally isolated from potato fields in the Leningrad region in 2021 and identified as mating type A2, was used for inoculation due to its high aggressiveness, confirmed in preliminary detached leaf assays [17]. A sporangial suspension at a concentration of 5 × 10^4^ sporangia/mL was prepared in sterile distilled water. Mycelium from cultures grown on rye agar was added to the suspension, which was incubated at 4–5 °C for 2–3 h before inoculation. Plants were inoculated by spraying the aerial parts with 300 µL of the sporangia suspension per plant, 24 h after peptide application (preventive treatment). The sporangia deposition was carried out under microscopic observation to confirm accurate inoculation.

### 2.3. Assessment of Disease Severity

Disease severity was assessed using the 1–9 Malcolmson composite scale, a semi-quantitative visual scoring system developed at the Scottish Crop Research Institute, which is commonly used in potato late blight studies. This scale evaluates the size of necrotic spots on leaves and overall amount of necrotic tissue per plant from 1 (highly susceptible/high symptom severity) to 9 (highly resistant/no symptoms) [18]. Disease symptoms were scored on the Malcolmson scale by an independent technical specialist who was blinded to the treatment groups.

### 2.4. Peptide Treatment

The synthetic peptide StPep1 was applied as a preventive foliar spray 24 h prior to *P. infestans* inoculation. Two concentrations were tested: 100 nM and 10 nM, with the addition of Neon (a non-ionic surfactant, 1:2000 dilution) to enhance adhesion. Each plant received approximately 300 µL of spray solution, ensuring thorough coverage of aerial parts.

### 2.5. Environmental Conditions

All experiments were conducted in a controlled climate chamber under the following conditions: 19–20 °C during the day and 10–11 °C at night, with relative humidity maintained at 80% during the day and 90% at night, a 14 h light/10 h dark photoperiod, and a light intensity of 60 µmol m^−2^ s^−1^.

### 2.6. Experimental Design

This study aimed to evaluate the preventive effect of the synthetic peptide StPep1 on *S. tuberosum* cv. Gala resistance to *P. infestans*. Plants were treated with StPep1 at 100 nM or 10 nM, applied 24 h before pathogen inoculation. Disease severity was evaluated 7 days post-inoculation. Five experimental groups were included in the study: (1) negative control—plants sprayed with sterile distilled water containing the surfactant Neon (1:2000 dilution), without *P. infestans* inoculation; (2) positive control 1—plants inoculated with *P. infestans* without Neon application; (3) positive control 2—plants sprayed with Neon (1:2000 dilution) without peptide treatment, followed by *P. infestans* inoculation 24 h later; (4) StPep1 (10 nM)—plants sprayed with 10 nM StPep1 formulated with Neon (1:2000 dilution), followed by *P. infestans* inoculation 24 h later; and (5) StPep1 (100 nM)—plants sprayed with 100 nM StPep1 formulated with Neon (1:2000 dilution), followed by *P. infestans* inoculation 24 h later. All plants were inoculated simultaneously across experimental groups.

### 2.7. Peptide Synthesis and Characterization

The 23-amino acid peptide StPep1 (sequence: ATERRGRPPSRPKVGSGPPPQNN) was synthesized using solid-phase peptide synthesis (SPPS) following the N-α-FMOC protocol. The crude product was purified by high-performance liquid chromatography (HPLC). Peptide identity and ≥95% purity were confirmed by reversed-phase HPLC and MALDI-TOF/TOF mass spectrometry, with further confirmation by Orbitrap nano-liquid chromatography tandem mass spectrometry (nano-LC-MS/MS).

### 2.8. Statistical Analyses and Graphical Representation

Statistical analyses (one-way ANOVA followed by Tukey’s post hoc test) and graphical representation of the data (boxplots with mean ± SE, *n* = 5 from three independent experiments) were performed using Python 3 with the NumPy 2.0.2, Matplotlib 3.9.4, and Statsmodels 0.14.5 libraries.

## 3. Results and Discussion

To explore the potential of the synthetic peptide StPep1 as a bioprotective agent for agricultural use, we employed foliar spraying as the most practical and widely applicable method of plant treatment. This approach enables direct application of bioactive compounds to the aerial parts of plants, facilitating rapid uptake and response. Initial experiments were conducted on potato (*S. tuberosum* cv. Gala) using a concentration of 100 nM StPep1, followed by testing at a lower concentration of 10 nM to evaluate efficacy at minimal dosages. The choice of nanomolar concentrations was guided by previous studies with Pep peptides from Arabidopsis, maize and potato [8,19,20], where protective activity was demonstrated at 10–100 nM, thereby providing the rationale for our experimental design.

**Figure 1:**
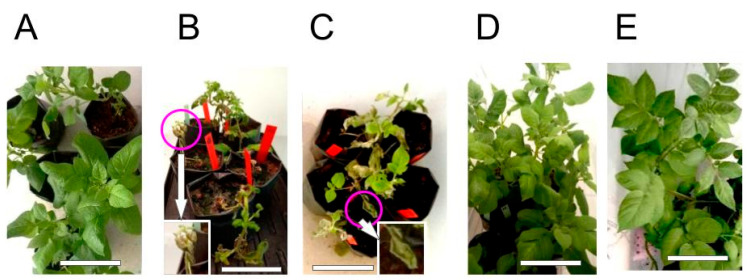
Visual appearance of *Solanum tuberosum* cv. Gala plants on day 7 after inoculation with *Phytophthora infestans* strain. (**A**) Negative control—healthy plants sprayed with sterile distilled water containing the surfactant Neon (1:2000 dilution), not inoculated and not treated with peptide. (**B**) Positive control 1—plants inoculated with *P. infestans* without peptide treatment and without Neon application, showing severe disease symptoms, extensive leaf damage, and markedly reduced leaf mass. (**C**) Positive control 2—plants sprayed with Neon and inoculated with *P. infestans* without peptide treatment, showing severe disease symptoms, including severe plant stunting and necrotic leaves. The pathogenic symptoms observed in both positive controls are characteristic of the *P. infestans* VZR ViR21a strain. Leaves displaying characteristic brown and shriveled symptoms of late blight are highlighted by purple circles, connected by white arrows to their corresponding magnified views in the inset panels in (**B**,**C**). (**D**) Preventive treatment with StPep1 at 100 nM, applied 24 h before inoculation—plants remain healthy with minimal symptoms. (**E**) Preventive treatment with StPep1 at 10 nM, applied 24 h before inoculation—plants exhibit strong protection comparable to the negative control. Images are provided for illustrative purposes to demonstrate overall plant condition, including growth and leaf mass, and are not intended for assessing disease severity based on necrotic lesions. Scale bar: 10 cm.

**Figure 2:**
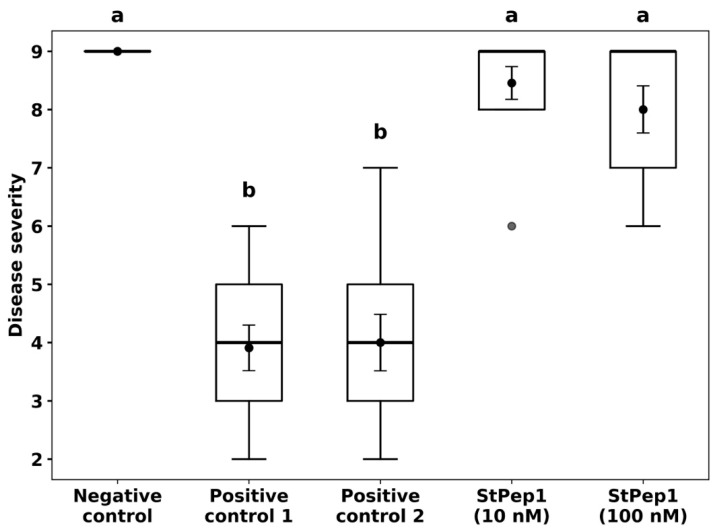
Resistance to *Phytophthora infestans* in *Solanum tuberosum* cv. Gala plants following preventive StPep1 treatment (24 h prior to inoculation). Disease severity (Malcolmson scale, 1 = highly susceptible, 9 = highly resistant) is shown as mean ± SE (black points with error bars inside the boxes), with boxplots representing the interquartile range and bold dots indicating outliers; *n* = 5 from three independent experiments. **Negative control**—plants sprayed with sterile distilled water containing the surfactant Neon (1:2000 dilution), without *P. infestans* inoculation; **Positive control 1**—plants inoculated with *P. infestans* without Neon application;  **Positive control 2**—plants sprayed with Neon (1:2000 dilution) without peptide treatment, followed by *P. infestans* inoculation 24 h later; **StPep1 (10 nM)** and **StPep1 (100 nM)**—plants sprayed with 10 nM and 100 nM StPep1, respectively, each formulated with Neon (1:2000 dilution), followed by *P. infestans* inoculation 24 h later. Different letters indicate statistically significant differences (*p* < 0.01, Tukey HSD). StPep1 treatments differ significantly from the positive controls, while no significant differences were observed between the 10 nM and 100 nM StPep1 treatments.

Preventive treatment of *S. tuberosum* plants with the synthetic peptide StPep1, applied 24 h prior to inoculation with *P. infestans*, conferred a strong protective effect against late blight under controlled climate chamber conditions (Figure 1). Visual assessment on day 7 post-inoculation revealed that plants treated with StPep1 at both 100 nM and 10 nM concentrations displayed only minimal disease symptoms if any. In both cases, the overall appearance of the treated plants closely resembled that of the negative control group (uninoculated plants) showing healthy green leaves with minimal disease symptoms, while the positive control groups (inoculated with *P. infestans* without peptide treatment) exhibited severe plant stunting, leaf and stem necrosis and a markedly reduced foliage compared to the treated plants, which were typical of VZR ViR21a strain (Figure 1 and Figure 2). Statistical analysis of disease severity, assessed using the Malcolmson 1–9 composite scale, indicated that both StPep1-treated groups were significantly different from the positive controls, whereas no significant differences were observed between either treatment and the negative control. Furthermore, the difference between the 10 nM and 100 nM StPep1 treatments was not significant, suggesting comparable protective effects at both concentrations (Figure 2). These results indicate that foliar application of StPep1 prior to oomycete infection effectively enhances resistance to *P. infestans*, even at low nanomolar concentrations.

Our findings demonstrate that the short, 23-amino acid potato elicitor peptide StPep1 acts as a highly effective bioprotective agent at nanomolar concentrations when applied by foliar spraying. Its protective effect is rapidly manifested within 24 h, reflecting a systemic induction of immune responses at the whole-organism level. This systemic action, combined with its exceptional low-dose efficacy, underscores the cost-effectiveness and practical potential of StPep1 for agricultural application. Beyond its demonstrated efficiency against *P. infestans*, these results highlight the promise of StPep1 as a novel protective agent that may also strengthen plant resistance to viral infections (our unpublished data), thereby opening new directions for translational research and crop protection strategies.

Elicitor peptides such as StPep1 from potato are regarded as promising tools for enhancing crop resilience through natural defense activation [9,20]. This perspective is grounded in the discovery of specific receptors and elucidation of molecular signaling pathways that are initiated by Pep peptides. The biological activity of Peps is mediated through its interaction with the leucine-rich repeat receptor kinase PEPR and is regulated by key stress-related signaling molecules, including methyl jasmonate and methyl salicylate. In Arabidopsis, binding of AtPep1 to PEPR1 activates a signaling cascade that leads to the production of reactive oxygen species (ROS) and the transcriptional activation of numerous defense-related genes, thereby enhancing the plant’s resistance to a broad range of biotic stresses [7,8,10,20,21]. Perception of Peps has been shown to enhance resistance in Arabidopsis, maize, and rice against diverse biotic challenges, including bacterial and fungal pathogens as well as insect herbivores [8,22,23,24], and also to confer tolerance to abiotic stresses such as high salinity [25,26]. These findings support the concept that Peps function as amplifiers of pattern-triggered immunity (PTI). Moreover, transcriptome analyses suggest that the role of Peps extends beyond defense signaling, implicating them in broader stress adaptation processes and even in the regulation of plant development [27,28]. Thus, Pep peptides represent a versatile class of signaling molecules with multifaceted functions in plant biology, making them valuable targets for translational applications in crop protection and improvement.

## 4. Conclusions

Our study identifies StPep1 as a promising elicitor peptide that can complement existing pathogen management practices based on pesticides and microbial biocontrol agents. By activating natural plant immunity, it supports more sustainable strategies for crop defense. In our experiments, preventive foliar application of StPep1 at a concentration as low as 10 nM significantly enhanced the resistance of *S. tuberosum* cv. Gala to *P. infestans* under controlled climate chamber conditions. Notably, the nanomolar effective concentration of StPep1 is several orders of magnitude lower than the millimolar or high-micromolar concentrations typically required for chemical fungicides, insecticides, and herbicides. This effective low-dose response highlights both the minimal environmental footprint and the cost-efficiency of StPep1 (currently estimated at approximately 10 USD per acre even at small-scale peptide synthesis), making it a promising candidate for small-scale agricultural trials. These findings support the applied potential of StPep1 as a novel bioprotective agent for potato cultivation. Building on this work, small-scale field trials are warranted to further assess its efficacy and explore its integration into standard agricultural formulations for preventive plant treatment.

## Data Availability

The original contributions presented in this study are included in the article. Further inquiries can be directed to the corresponding author.
